# Self-care practices during pregnancy and associated factors among pregnant mothers who attended and underwent follow-up at public health facilities in Jimma Town, Oromia, Southwest Ethiopia, in 2023

**DOI:** 10.3389/fgwh.2025.1567973

**Published:** 2025-11-26

**Authors:** Negash Sherif, Ayanos Taye, Temesgen Geta Hardido, Christian Kebede Gadabo, Dawit Tesfaye, Tamirat Beyene Gerete

**Affiliations:** 1 School of Nursing, Institute of Health Sciences, Jimma University, Jimma, Ethiopia; 2 School of Nursing, College of Health Sciences and Medicine, Wolaita Sodo University, Wolaita Sodo, Ethiopia; 3 School of Midwifery, College of Health Sciences and Medicine, Wolaita Sodo University, Wolaita Sodo, Ethiopia

**Keywords:** self-care, practice, pregnant women, Jimma, Ethiopia

## Abstract

**Background:**

Self-care practices during pregnancy significantly influence individuals, families, and communities by fostering health, preventing illness, sustaining wellbeing, and managing health challenges, irrespective of whether there is assistance from healthcare professionals. Recognizing the importance of self-care interventions facilitates the formulation of novel and equitable strategies to enhance access to sexual and reproductive health services, which may also lead to a reduction in healthcare costs by minimizing the need for travel to medical facilities. However, studies on self-care practices during pregnancy are limited in Ethiopia. Therefore, this study aims to assess self-care practices and associated factors among pregnant women who attended public health facilities in the town of Jimma, Oromia, Southwest Ethiopia, in 2023.

**Methods:**

A facility-based cross-sectional study was conducted from 1 to 30 May 2023, on 317 study participants who were selected by simple random sampling techniques and interviewed using structured questionnaires. The data were entered into EpiData version 3.1 and exported to SPSS version 26 for analysis. Descriptive statistics were computed to summarize the data and describe the study participants. Bivariate and multivariate logistic regression analyses were conducted to identify the factors associated with the outcome variable; a 95% confidence interval and a *P*-value of < 0.05 were taken as the cutoff points to determine whether there was a statistically significant association. Descriptive statistics were presented using figures, tables, and text.

**Results:**

The findings of the current study revealed that nearly half (49.52%) of the pregnant mothers demonstrated good self-care practices. In addition, the following factors were significantly associated with the self-care practices of pregnant mothers: history of abortion (adjusted odd ratio (AOR): 0.44, 95% CI = 0.2003–0.952, *P* = 0.037), knowledge (AOR: 5.205, 95% CI = 2.679–10.115, *P* = 0.001), attitude (AOR: 6.034, 95% CI = 3.217–11.317, *P* = 0.002), and social support (AOR: 1.255, 95% CI = 1.668–2.36, *P* = 0.048).

**Conclusion and recommendation:**

The findings suggested that only approximately half of the women followed good self-care practices during pregnancy. It is recommended that pregnant mothers should possess the relevant knowledge about the recommended practices to be followed and activities to be avoided during pregnancy.

## Introduction

Self-care practices encompass a process through which individuals and families utilize knowledge and beliefs, self-regulation, skills, abilities, and social facilitation to attain health outcomes, particularly during pregnancy (1). Self-care practices during pregnancy play a crucial role in promoting health, preventing disease, facilitating self-medication, maintaining health, and managing illness and disability, with or without the assistance of a healthcare provider (2, 3).

Pregnant women need effective self-care practices to enhance pregnancy outcomes and sustain their health, which can be accomplished by understanding lifestyle-related risk factors. Nevertheless, in low- and middle-income countries, the obstacles presented by low socioeconomic status and insufficient health literacy significantly impede the capacity to engage in self-care (4).

Unhealthy self-care practices during pregnancy can negatively impact the health of women and the developmental status of their children. This can result in numerous physical and psychological consequences, as well as an increased risk of birth defects, miscarriage, or preterm birth (5–7). Heightened psychological stress and prenatal depression can disrupt homeostasis and weaken the immune system, thereby increasing the likelihood of complications such as preeclampsia, preterm birth, or miscarriage (8).

Pregnant women primarily seek consultation from health professionals only when they are critically ill. However, they often engage in self-medication, including the use of traditional treatments or herbal remedies, which are frequently suggested by their mothers or mothers-in-law to prevent and alleviate certain symptoms (9). Furthermore, pregnant women may take over-the-counter medications without the guidance and counseling of health personnel, exposing themselves to potential risks (10). On their part, healthcare providers exhibit a lack of focus on delivering care and minimizing obstacles to enhance the self-care of pregnant women; also, there is inadequate communication between healthcare providers and expectant mothers (2).

Numerous studies have revealed that pregnant women frequently neglect proper personal hygiene practices and seldom seek assistance from healthcare providers during their pregnancy. Moreover, maternal health literacy is identified as a critical barrier to self-care, leading to a decrease in confidence regarding health management. This diminished confidence negatively impacts self-care behaviors throughout pregnancy. In addition, factors such as limited knowledge, a failure to identify oneself as part of a high-risk group, and a lack of guidance on health practices exacerbate this problem (9–11).

Self-care during pregnancy is a primary objective of health programs; however, significantly less emphasis is placed on health-promoting self-care interventions during this period (12). There is limited research on self-care practices among health pregnant women in Ethiopia. In addition, modern lifestyles and access to health information may influence the current self-care practices among pregnant women. Therefore, an investigation was required into self-care behaviors among pregnant women on the aspect of maintaining health and wellbeing. Consequently, this study aims to assess self-care practices and their associated factors among pregnant women attending public health facilities in the town of Jimma, Oromia, Southwest Ethiopia, in 2023.

## Methods and materials

### Study area and period

The study was conducted in public health facilities situated in Jimma Town, Oromia, Ethiopia, from 1 to 30 2023.Jimma Town acts as the administrative hub of the Jimma zone and is located approximately 356 km from Addis Ababa, the capital city of Ethiopia, in the southwestern part of the nation. The town consists of 17 kebeles, which are managed by the municipal government. Its geographical coordinates are approximately 7°40′N latitude and 36°50′E longitude. Jimma Town has an overall population of 207,573, amounting to a population density of 4,151.46 individuals per square kilometer. Within Jimma Town, there exists two public hospitals—Jimma University Medical Center and Shanen Gibe Hospital—in addition to four health centers (13).

### Study design

A facility-based cross-sectional study design was devised.

### Population

#### Source population

The source population included all pregnant women who had antenatal care (ANC) follow-ups scheduled at selected public health facilities in Jimma Town.

#### Study population

The study population included all selected pregnant women who were attending ANC follow-up at selected public health facilities in Jimma Town.

### Sample size determination and sampling technique

#### Sample size determination

The sample size was estimated by using the following single proportion formula.

Where *n* is the initial sample size required, *p* is the magnitude of good dietary practice among pregnant women in Mizan-Aman Town, estimated at = 25.1% (14), *d* is the margin of sampling error tolerated with a 95% confidence level, and Nf is the final sample size.

$$n=\frac{Z\;{\left(\alpha /2\right)}^{2}p\;(1-p)}{d^{2}}=\frac{{\left(1.96\right)}^{2}\times 0.25(1-0.25\;)}{{\left(0.05\right)}^{2}}=288$$

By adding a 10% non-response rate, the final sample size amounted to 317 subjects.

#### Sampling technique

**Figure 1:**
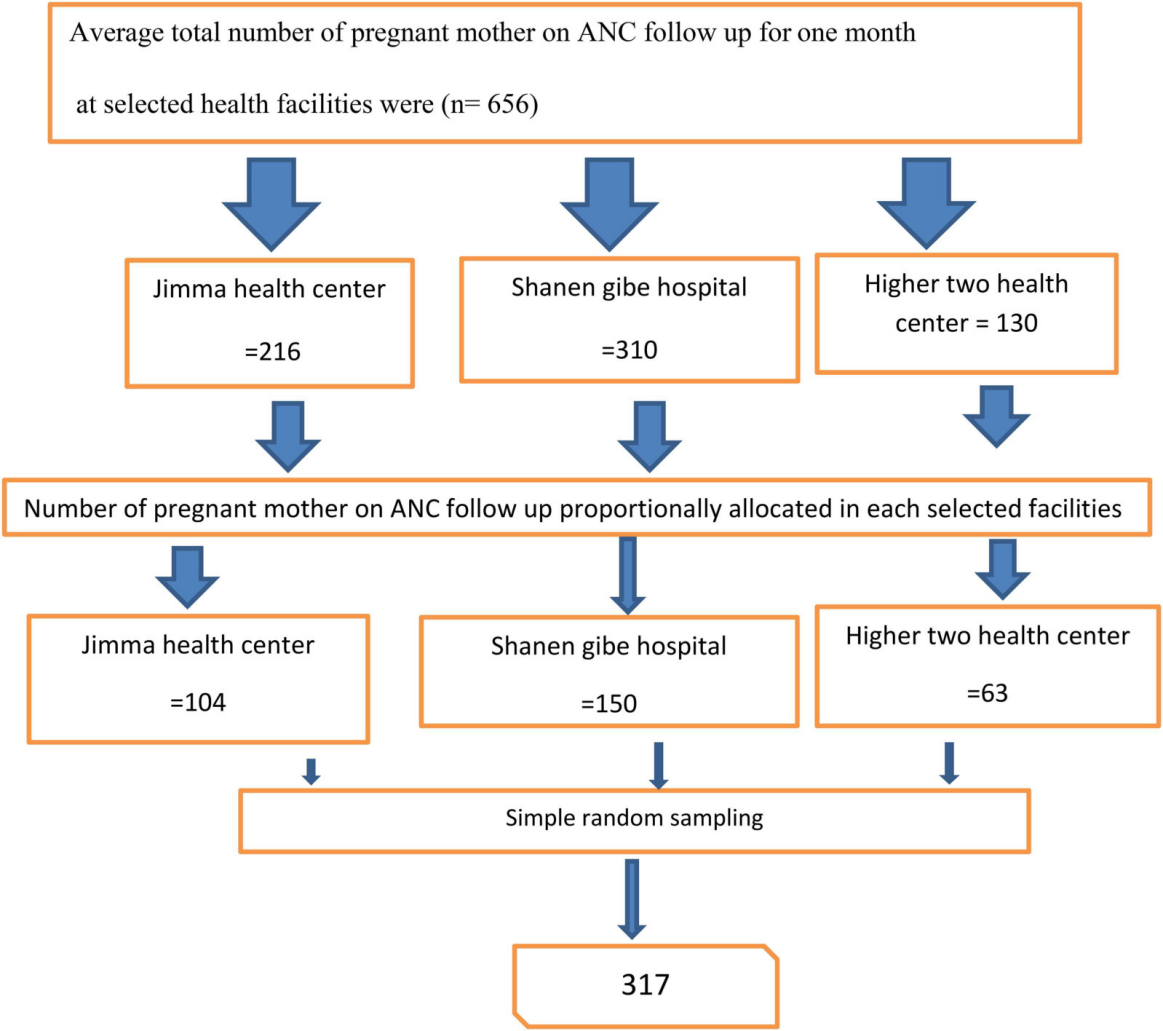
Schematic representation of the sampling technique and procedure to assess self-care practices and associated factors among pregnant women attending ANC follow-up at public health facilities in Jimma Town, Oromia, Ethiopia, in 2023.

Initially, three health facilities—Shanen Gibe Hospital, Jimma Health Center, and Higher Two Health Center—were selected from a total of six public health facilities in Jimma Town using a simple random sampling technique through the lottery method. Subsequently, the average number of women who attended ANC follow—up sessions at each selected health facility in Jimma Town over the past three months was recorded. Based on this information, the required sample size was proportionally distributed among the selected facilities, taking into account the average number of women participating in ANC follow—up at each site. Participants were chosen using the simple random sampling technique. Finally, eligible individuals from the public health facilities were recruited for the study (Figure 1).

### Study variables

The dependent variable of this study was self-care practice. The independent variables of this study included age, level of education, occupation, marital status, number of family members, gestational age, gravidity, parity, history of abortion, family support, support from relatives, knowledge, and attitude toward recommended health practices during pregnancy.

### Operational definitions

Self-care practices during pregnancy refer to behaviors that underscore recommended and prohibited practices including proper nutrition, avoiding harmful substances, drug intake/medication, regular exercise, follow-up medical visits, and the required number of visits (15).

Good self-care practice: participants who answered self-care practice questions correctly and scored greater than or equal to the mean score (15).

Poor self-care practice: participants who answered self-care practice questions correctly, but scored below the mean score (15).

Good knowledge: participants who answered knowledge-based questions correctly and scored greater than or equal to the mean score (11).

Poor knowledge: participants who answered knowledge-based questions correctly but scored below the mean score (11).

Favorable attitude: participants who answered attitude-related questions correctly and scored greater than or equal to the median score (16).

Unfavorable attitude: participants who answered attitude-related questions correctly, but scored below the median score (16).

Good social support: participants who answered social support questions correctly and scored greater than or equal to the mean score (17).

Poor social support: participants who answered social support questions correctly, but scored below the mean score (17).

### Data collection process and technique

#### Data collection instruments

Thew data collection tools for this study were adapted after a review of different relevant studies (11, 15–18). Data were collected using a standardized, structured questionnaire administered by interviewers, adapted from various sources. All questions were translated into two local languages (Afaan Oromo and Amharic) by experts proficient in both languages, and subsequently translated back into English to verify consistency. The questionnaire consisted of five sections totaling 62 items. The first section included seven items related to the sociodemographic characteristics of the respondents, which were addressed accordingly. The second section focused on obstetric factors, comprising four items. The third section examined factors related to individual pregnant women, including their knowledge and attitudes. The fourth section included six items pertaining to the social support available to pregnant women.

Finally, the last section contained 13 items related to maternal self-care practices during pregnancy, which included nutrition (four items), substances (three items), drug intake (one item), physical activity (one item), and follow-up and number of visits (four items), all of which were employed to measure the outcome variable of the study.

#### Data collection techniques

Data were collected via face-to-face interviews using a structured questionnaire. Pregnant women who had at least one ANC follow-up visit scheduled during the data collection period were interviewed in person using the questionnaire. Three BSC midwives were selected based on their previous experience in data collection. Clinical data relating to the patients were extracted from the mother chart cards using an extraction checklist.

### Quality control

The questionnaire that was developed in English was translated into two local languages—Afaan Oromo and Amharic—by skilled multilingual translators. A pretest of the questionnaires was conducted on 5% of the sample size—which included 16 study participants at Yabu Health Center—to evaluate the data for clarity, feasibility, applicability, understandability, reliability, and coherence. This pretest also aimed to identify potential challenges that could arise during data collection and to estimate the time required to complete the questionnaire items. Following the feedback received, necessary revisions were implemented. Certain items were clarified, ambiguous words or phrases were removed, and terms that are locally recognized and comparable were utilized. The results from the pretest indicated a Cronbach's alpha of 0.728 for knowledge, 0.783 for attitude, 0.708 for social support, and 0.85 for self-care practices.

An orientation session lasting 2–3 h was provided to the data collectors to ensure that all individuals involved had a uniform understanding of the study instrument and adhered to the same interview protocols. The principal investigator conducted continuous follow-ups throughout the data collection phase. In addition, daily reviews of all completed questionnaires were performed by the principal investigator to guarantee the completeness and consistency of the data collected.

### Data processing and analysis

After data collection, the data were coded and entered into a computer using the Epi-data 3.1 statistical program. The data were then exported to SPSS version 26 for further analysis. Descriptive statistics were calculated. A bivariate logistic regression model was run to assess the association between independent and dependent variables. Candidate variables for the final model were identified using bivariate logistic regression at a *p*-value of less than 0.25. Then, multivariate logistic regression was performed to analyze the independent effect of each explanatory variable on the dependent variable at a *p*-value of 0.05. An AOR with a 95% confidence interval was used to show the magnitude of association.

The model fitness for the variables was assessed using the Hosmer and Lemeshow goodness-of-fit test at *P* > 0.05. A *P*-value of <0.05 was considered to be the cutoff point to declare a statistically significant association between independent and dependent variables. The odds ratio was calculated to determine the strength of association between the two variables. Finally, the results were presented using text, tables, and graphs.

### Ethical approval

This research was conducted in accordance with the Helsinki Declaration. Ethical approval for this study was obtained from the Ethical Review Committee of Jimma University with the reference number JUIH/IRB/413/2023. Subsequently, a letter was submitted to Jimma Town public health facilities to obtain permission before the actual data collection.

## Results

### Sociodemographic characteristics

Out of a total sample size of 317, 311 pregnant women were included in the present study, resulting in a response rate of 98.1%. The findings revealed that over half (58.5%) of the participants fell between the ages of 20 and 30 years. In terms of educational attainment, only 7.7% of the participants had completed college-level education or higher. The majority of participants (90.4%) were married, and nearly half (43.1%) identified as housewives. In addition, a significant proportion of participants (78.8%) reported having more than two family members, and 85.5% were residents of urban areas (Table 1).

**Table 1:** Sociodemographic characteristics of pregnant women attending public health facilities in Jimma Town, Oromia, Southwest Ethiopia, in 2023.

Variables	Category	Frequency	Percent
Age of mothers in year	15–20	30	9.6
	20–30	182	58.5
	>30	99	31.8
Level of education	Illiterate	58	18.6
	Can read and write	36	11.6
	Primary education	125	40.2
	Secondary education	68	21.9
	College and above	24	7.7
Marital status	Single	6	1.9
	Married	281	90.4
	Divorced	14	4.5
	Widowed	10	3.2
Occupation	Housewife	134	43.1
	Employed	42	13.5
	Merchant	61	19.6
	Daily laborer	56	18.0
	Others	18	5.8
Number of family members	=2	66	21.2
	>2	245	78.8
Monthly household income in ETB	<1,500	24	7.7
	1,500–5,000	157	50.5
	>5,000	130	41.8
Resident	Rural	45	14.5
	Urban	266	85.5

### Obstetric factors

The finding showed that 42.4% of the respondents were in the third trimester of pregnancy; about half (48.9%) of the respondents had a gravidity of three or more, and only 18% had a history of abortion (Table 2).

**Table 2:** Obstetric factors of pregnant women attending public health facilities in Jimma Town, Oromia, Southwest Ethiopia, in 2023.

Variables	Category	Frequency	Percent
Gestational age	First trimester	68	21.9
	Second trimester	111	35.7
	Third trimester	132	42.4
Number of gravida	One	62	19.9
	Two	97	31.2
	Three or more	152	48.9
Number of para	Null	74	23.8
	One	87	28.0
	Two	48	15.4
	Three or more	102	32.8
Do you have a history of abortion?	Yes	56	18.0
	No	255	82.0

### Knowledge-related factors

**Figure 2:**
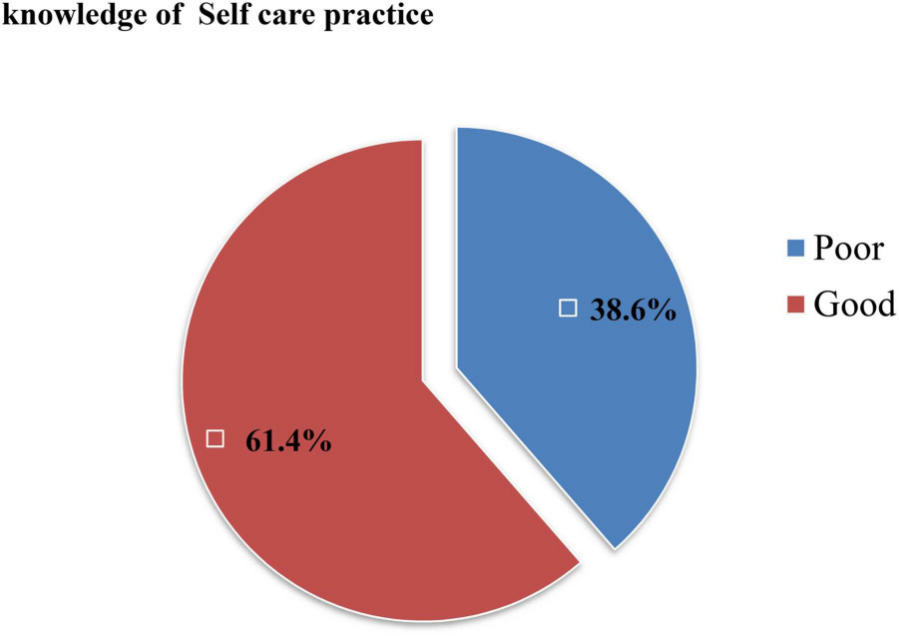
Knowledge of self-care practices among pregnant women attending public health facilities in Jimma Town, Oromia, Southwest Ethiopia, in 2023.

With regard to the knowledge about the recommended practices to be followed and the precautionary steps to be taken during pregnancy, more than half (61.41%) of the respondents reported good knowledge about these (Figure 2).

The majority (87.5% and 60.1%, respectively) of the respondents correctly noted that drinking alcohol is not recommended during pregnancy and smoking during pregnancy has a harmful effect on birth outcomes. The findings suggest that 58.8% of the respondents noted the importance of physical activity during pregnancy, 75.9% understood the importance of taking vitamins and minerals, and 68.5% recognized the importance of calcium intake during pregnancy (Table 3).

**Table 3:** Knowledge of pregnant women attending public health facilities in Jimma Town, Oromia, Southwest Ethiopia, in 2023.

Variables	Response	Frequency	Percent
Drinking alcohol is recommended during pregnancy	Yes	39	12.5
	No	272	87.5
Smoking during pregnancy has effect on birth outcome	Yes	187	60.1
	No	124	39.9
Does exposure to cigarette smoke have an effect on pregnancy?	Yes	140	45.0
	No	171	55.0
Is it recommended to use addictive substances such as drugs during pregnancy?	Yes	56	18.0
	No	255	82.0
Is using traditional medicine recommended during pregnancy?	Yes	205	65.9
	No	106	34.1
Is it important to engage in physical activity during pregnancy?	Yes	183	58.8
	No	128	41.2
Is taking vitamins and minerals during pregnancy important?	Yes	236	75.9
	No	75	24.1
Is taking iron and folic acid important during pregnancy?	Ye	266	85.5
	No	45	14.5
Is it important to take calcium during pregnancy?	Yes	213	68.5
	No	98	31.5
Is it important to eat fiber-containing vegetables during pregnancy?	Yes	270	86.8
	No	41	13.2
Is it important to consult health workers about maternity care during pregnancy?	Yes	275	88.4
	No	36	11.6
Is it important to discuss the effects of medication on pregnancy with health professionals?	Yes	300	96.5
	No	11	3.5
Is it important to follow ANC check-up according to the schedule?	Yes	308	99.0
	No	3	1.0

### Self-care practices during pregnancy

**Figure 3:**
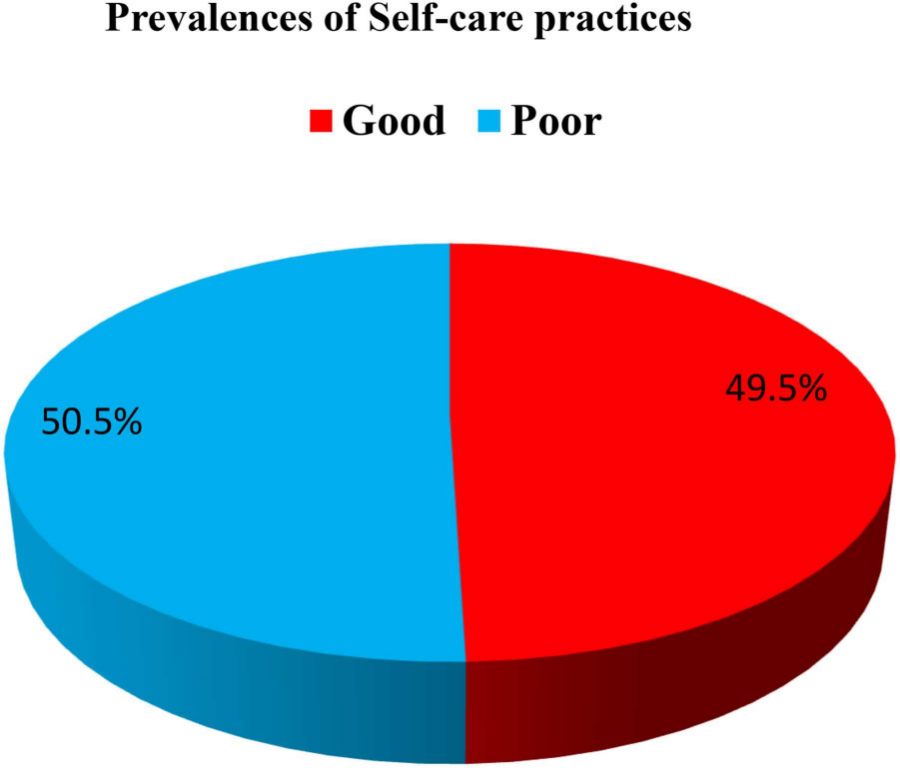
Prevalence of self-care practices among women attending public health facilities in Jimma Town, Oromia, Southwest Ethiopia, in 2023.

Prior to categorization, the self-care practice scores had a median of 43.00, a range of 29 (30–59), and a mean score of 43.69. The self-care practices were categorized based on the mean score into good and poor self-care practices: patients who had a mean score of 43.69 and above on self-care practice questions were categorized as having good self-care practices, whereas patients who scored below the mean of 43.69 on these questions were identified as having poor self-care practices. The findings indicated that 157 (50.48%) of the expectant mothers exhibited poor self-care practices, while 154 (49.52%) demonstrated good practices (Figure 3).

A significant majority of the participants (83.3%) reported refraining from alcohol consumption during their pregnancy. Almost all respondents (94.9%) stated that they did not smoke, although 14.1% were frequently exposed to secondhand smoke. The frequent use of traditional medicine during pregnancy was reported by 41.2% of the participants, with an additional 25.1% using it occasionally. Only 9.3% of the study population engaged in regular physical activity, and 7.5% consistently consumed vitamins and minerals. Furthermore, a mere 11.3% adhered to the recommended intake of iron and folic acid. In addition, only 17.7% of respondents sought guidance from healthcare professionals regarding maternity care, while 11.6% discussed the implications of medication use during pregnancy with health experts. Notably, approximately half of the participants (51.8%) reported that they never missed their scheduled follow-up appointments (Table 4).

**Table 4:** Self-care practices of pregnant women attending public health facilities in Jimma Town, Oromia, Ethiopia, in 2023.

Specific activities	Always *N* (%)	Most of the time *N* (%)	Some of the time *N* (%)	Rarely *N* (%)	Never *N* (%)	Mean and SD
How often did you consume alcohol?	0	2 (0.6)	25 (8)	25 (8)	259 (83.3)	4.74 ± 0.63
How often did you smoke during pregnancy?	0	1 (0.3)	3 (1)	12 (3.9)	295 (94.9)	4.93 ± 0.32
How often were you exposed to somebody smoking?	1 (0.3)	44 (14.1)	128 (41.2)	32 (10.3)	106 (34.1)	3.64 ± 1.10
Have you used addictive substances such as drugs?	0	3 (1)	12 (3.9)	56 (18)	240 (77.2)	4.71 ± 0.58
Have you used traditional medicine?	14 (4.5)	128 (41.2)	78 (25.1)	10 (3.2)	81 (26)	3.05 ± 1.29
Have you missed doing physical activity/week?	29 (9.3)	42 (13.5)	170 (54.7)	41 (13.2)	29 (9.3)	3.00 ± 1.01
Have you missed taking vitamins and minerals?	103 (33.1)	83 (26.7)	85 (27.3)	17 (5.5)	23 (7.4)	2.27 ± 1.19
Have you missed taking iron and folic acid as recommended?	22 (7.1)	61 (19.6)	149 (47.9)	44 (14.1)	35 (11.3)	3.03 ± 1.036
Have you missed taking calcium as recommended?	157 (50.5)	64 (20.6)	66 (21.2)	11 (3.5)	13 (4.2)	1.90 ± 1.11
Have you skipped eating fiber-containing vegetables/week?	17 (5.5)	49 (15.8)	204 (65.6)	27 (8.7)	14 (4.5)	2.91 ± 0.79
Have you consulted health workers on maternity care?	55 (17.7)	68 (21.9)	159 (51.1)	13 (4.2)	16 (5.1)	2.57 ± 0.99
Have you discussed the effects of medication on pregnancy with health professionals?	36 (11.6)	54 (17.4)	188 (60.5)	16 (5.1)	17 (5.5)	2.76 ± 0.92
Have you missed scheduled follow-up?	10 (3.2)	5 (1.6)	65 (20.9)	70 (22.5)	161 (51.8)	4.18 ± 1.03

### Attitude of respondents

**Figure 4:**
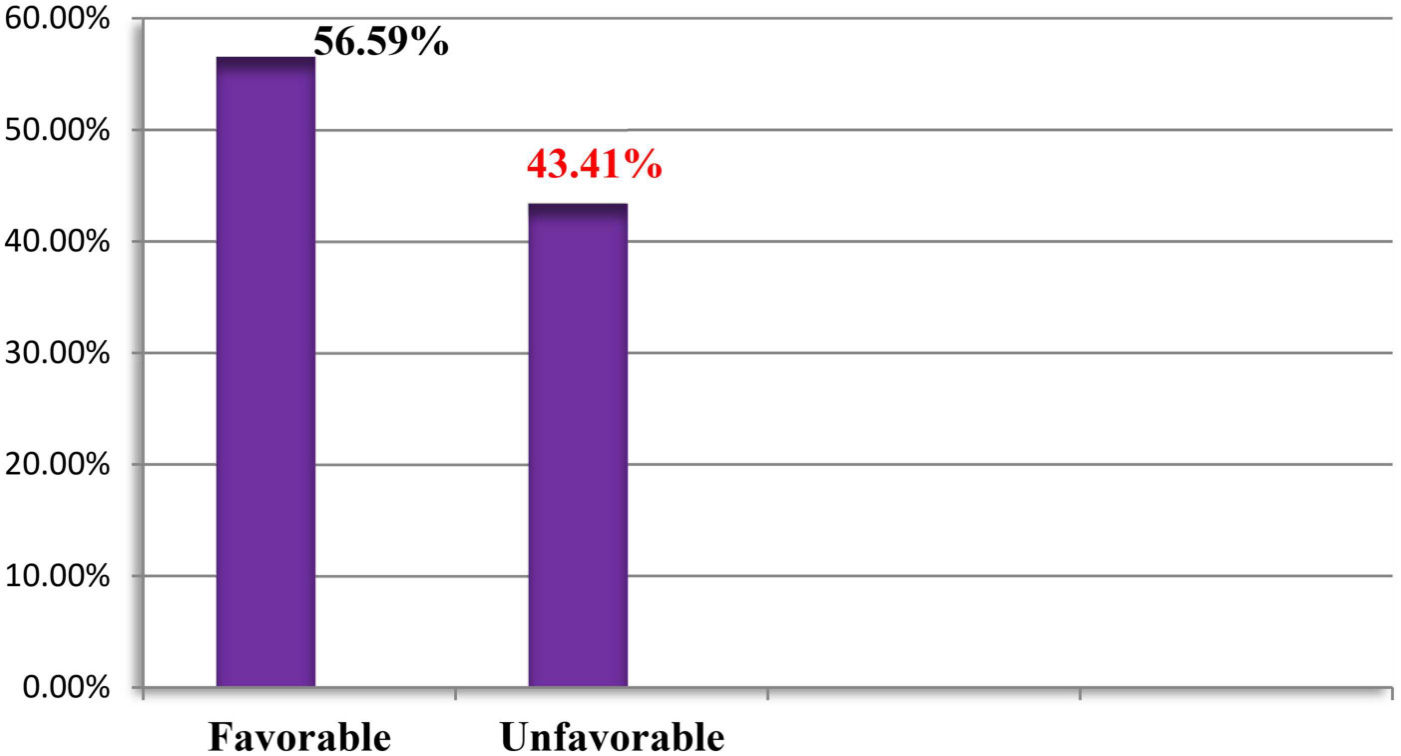
Attitude of pregnant women attending public health facilities in Jimma Town, Oromia, Ethiopia, in 2023.

The study findings suggested that more than half (56.59%) of the participants had favorable attitudes, while 43.41% of the respondents had unfavorable attitudes (Figure 4).

### Social support of respondents

**Figure 5:**
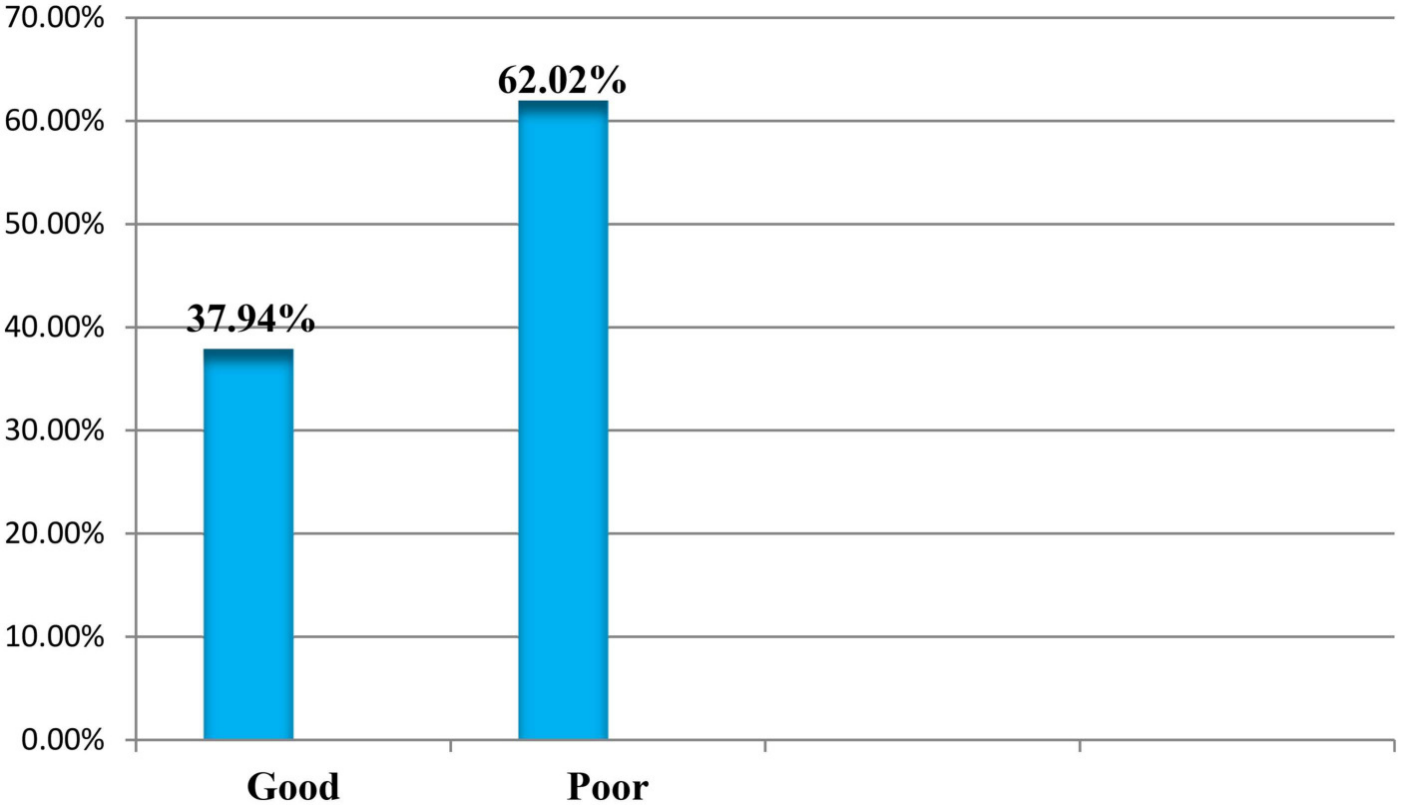
Social support received by pregnant women attending public health facilities in Jimma Town, Oromia, Ethiopia, in 2023.

Based on the study findings, most (62%) of the participants had poor social support, whereas only about 38% had good social support (Figure 5).

### Factors associated with self-care practices

In the bivariate analysis, the variables of age, occupation, monthly income, gravidity, parity, history of abortion, knowledge, attitude, and social support were candidates (*P* < 0.25) for multiple logistic regression.

The results of the multivariate analysis show that history of abortion, knowledge, attitude, and social support had significant association with the self-care practices of pregnant mothers.

Participants with a history of abortion were 56% less likely to follow good self-care practices (AOR: 0.44, 95% CI = 0.2003–0.952, *P* = 0.037). Participants with good knowledge were five times more likely of having good self-care practices (AOR: 5.205, 95% CI = 2.679–10.115, *P* = 0.001), compared with those with poor knowledge.

Those with a favorable attitude were six times more likely to follow good self-care practices (AOR: 6.034, 95% CI = 3.217–11.317, *P* = 0.002) than those with an unfavorable attitude. Women with good social support were 1.255 times more likely to follow good self-care practices (AOR: 1.255, 95% CI = 1.668–2.36, *P* = 0.048), compared with those with poor social support (Table 5).

**Table 5:** Multivariate analysis results of factors associated with self-care practices of pregnant women attending public health facilities in Jimma Town, Oromia, Southwest Ethiopia, in 2023.

Variable	Category	Self-care practice		COR	AOR (95% CI)	*P*-value
		Poor *N* (%)	Good *N* (%)			
Maternal age	>30	65 (65.7)	34 (34.3)	1	1
	15–20	81 (44.5)	101 (55.5)	2.384	1.002 (0.237, 4.241)	0.998
	20–30	11 (36.7)	19 (63.6)	3.302	1.194 (0.472, 3.019)	0.708
Occupation	Others	6 (33.3)	12 (66.7)	1	1	
	Housewife	64 (47.8)	70 (52.2)	.547	0.584 (0.142, 2.41)	0.457
	Employed	21 (50)	21 (50)	.500	0.452 (0.094, 2.41)	0.323
	Merchant	37 (60.7)	24 (39.3)	.324	0.271 (0.059, 1.251)	0.094
	Daily laborer	29 (51.8)	27 (48.2)	.466	0.428 (0.096, 1.911)	0.266
Monthly income	>5,000	57 (43.8)	73 (56.2)	1	1	
	<1,500	14 (58.3)	10 (41.7)	.558	0.626 (0.213, 1.842)	0.395
	1,500–5,000	86 (54.8)	71 (45.2)	.645	0.604 (0.331, 1.102)	0.100
Gravida	≥3	85 (55.9)	67 (44.1)	1	1	
	One	26 (41.9)	36 (58.1)	1.757	0.247 (0.037, 1.653)	0.149
	Two	46 (47.4)	51 (52.6)	1.407	0.393 (0.115, 1.344)	0.136
Para	≥3	69 (63.3)	33 (30.3)	1	1	
	Null	29 (39.2)	45 (60.8)	3.245	1.880 (0.841, 4.200)	0.124
	One	43 (49.4)	44 (50.6)	2.140	1.566 (0.709, 3.459)	0.267
	Two	16 (33.3)	32 (66.7)	4.182	2.309 (0.926, 5.757)	.073
Abortion	No	119 (46.5)	137 (53.5)	1	1	
	Yes	39 (69.7)	17 (30.3)	.375	0.44 (0.2003, 0.952)	0.037*
Knowledge	Poor	101 (84.2)	19 (15.8)	1	1	
	Good	56 (29.3)	135 (70.7)	12.815	5.205 (2.679, 10.115)	0.001*
Attitude	Unfavorable	111 (82.2)	24 (17.8)	1	1	
	Favorable	46 (26.1)	130 (73.9)	13.071	6.034 (3.217, 11.317)	0.002*
Social support	Poor	114 (59.1)	79 (40.9)	1	1	
	Good	43 (36.4)	75 (63.6)	2.517	1.255 (1.668, 2.36)	0.048*

* Significant variables at *P*-value < 0.05.

## Discussion

This study aimed to assess the self-care practices and associated factors among pregnant women attending public health facilities in Jimma Town, Oromia, Southwest Ethiopia, in 2023. In this study, almost half or 49.5% of the pregnant participants exhibited commendable self-care practices. These findings are consistent with those of a previous study conducted in Babylon City, which reported that 50% of expectant mothers demonstrated satisfactory self-care practices during the prenatal period (1).

In contrast, this research presents findings that differ to a slight extent from those of a study conducted in Hodeida City, Yemen, where 40.6% of pregnant women exhibited good universal self-care practices (19). However, the level of practice observed in the findings of this study is lower than that of the results reported in studies done in Province, Indonesia (76.4%) (12), and the northeastern region of Thailand (76.9%) (2). The potential reasons for this discrepancy may stem from variations in the sociodemographic and economic status of the study participants, differences in the study period and setting, or the different cutoff points employed to measure the variables.

Moreover, the current findings reveal that a significant majority (83.3%) of respondents abstained from alcohol consumption during their pregnancy. Almost all participants (94.9%) refrained from smoking; however, only 34.1% of women successfully avoided exposure to secondhand smoke, and 26% abstained from using traditional medicine without a prescription from a physician. These results are consistent with those of a study conducted in Vietnam, which reported that none of the pregnant women smoked during their pregnancy, although 13.4% continued to consume alcohol (3).

These findings are somewhat encouraging with regard to alcohol avoidance during pregnancy; however, it is crucial to highlight that only a small percentage of women refrained from alcohol consumption. Compared with the results of a study conducted in Vietnam, which indicated that only 78.7% of participants consistently avoided alcohol, 53.9% of pregnant women avoided secondhand smoke, and 71.7% refrained from using traditional medicine without a prescription from a physician, the results of the present study suggest a pressing need for enhanced awareness and education regarding related health risks (4). This indicates a significant exposure of pregnant women to secondhand smoke and a high prevalence of traditional medicine usage without prescriptions from a physician in the current study setting, which may be influenced by religious or cultural preferences.

Moreover, only 9.3% of the study participants regularly engaged in physical activity, while a mere 17.7% sought guidance from healthcare professionals concerning maternity care. Approximately half of the respondents (51.8%) reliably attended their follow-up appointments. These results are significantly lower than those from a previous study, which reported that 13% of pregnant women consistently engaged in physical exercise at least three times a week and around 66% regularly attended their scheduled prenatal appointments. The observed discrepancies may be linked to differences in socioeconomic status, levels of knowledge, or attitudes among respondents regarding the recommended activities for pregnant women (4).

The current study revealed that a history of abortion, knowledge, attitude, and social support significantly influenced the self-care practices of pregnant mothers.

Participants with good knowledge were 5.2 times more likely to practice good self-care behavior. This finding is supported by those of studies in Indonesia and the northeastern region of Thailand in which knowledge of self-care practices was indicated to be a determinant associated with self-care behavior (2, 12). This finding underscores the importance of knowledge, which should be encouraged by health institutions. Pregnant women with a history of abortion had a 56% decreased probability (AOR: 0.44, 95% CI = 0.2003–0.952, *P* = 0.037) of following good self-care practices. Although there is limited evidence, women who have had a history of abortion—due to a poor knowledge of lifestyle-related risk factors during pregnancy—are more likely to follow poor self-care practices.

Pregnant women with a favorable attitude were six times more likely to follow good self-care practices (AOR: 6.034, 95% CI = 3.217–11.317, *P* = 0.002) than those with an unfavorable attitude. This finding is consistent with those of the studies from Mizan-Aman Town (14). This may be justified by the fact that an individual who has a favorable attitude relating to a certain health phenomenon factually follows good self-care practices.

Finally, women with good social support were 1.255 times more likely to follow good self-care practices compared with those without good social support. In line with this study, studies conducted in Indonesia, Thailand, and Vietnam concluded that social support is a major driver of self-care practices (2, 4, 12). The implication of the present study is that pregnant women should receive social support from their spouse, family, and friends.

### Strengths and limitations

The strengths of this study include the utilization of primary data and the fact that this topic has not been previously researched in Ethiopia, making it a valuable resource for future reference. In addition, the use of a facility-based cross-sectional study design with robust statistical analyses—including multivariate logistic regression—can be considered a strength of this research.

### Limitation of the study

As this study employs a cross-sectional design, it does not establish a definitive cause-and-effect relationship between dependent and independent variables. In addition, due to the scarcity of literature on this subject within Ethiopia, comparisons with similar studies on pregnant women conducted in other nations were deemed necessary. Furthermore, the generalizability of the findings is limited as the research was exclusively conducted at health facilities in Jimma Town.

### Practical implications for maternal health programs

The implications of self-care practices during pregnancy for maternal health programs are significant, particularly in terms of promoting health, preventing disease, facilitating self-medication, and managing health and disabilities, with or without the assistance of healthcare providers. Recommendations for maternal health programs include educating pregnant women, early identification of potential health issues, enhancing nutritional status, and providing mental health support. Therefore, it is imperative to uphold and foster self-care practices during pregnancy.

## Conclusion and recommendation

The findings of this study indicated that only approximately half of the women followed good self-care practices during pregnancy.

Moreover, the findings identified the following as significant factors associated with the self-care practices of pregnant women: a history of abortion, knowledge, attitude, and social support.

Self-care practices during pregnancy should be encouraged and implemented at all levels, starting from the ministry of health to the primary healthcare unit.

Healthcare providers should implement health education programs at the health facility level to improve the knowledge and attitudes of pregnant women about recommended pregnancy self-care practices.

All pregnant mothers should be informed by their healthcare providers—during ANC follow-ups—about the complications of unsafe abortions.

Male involvement in antenatal care should be encouraged at the community level by health extension workers and community leaders.

Further research is needed to explore additional factors influencing self-care practices among pregnant women in local contexts.

## Data Availability

The raw data supporting the conclusions of this article will be made available by the authors without undue reservation.
